# Suspected lead poisoning in two captive cheetahs (*Acinonyx jubatus jubatus*) in South Africa, in 2008 and 2013

**DOI:** 10.4102/jsava.v86i1.1286

**Published:** 2015-08-13

**Authors:** Michelle A. North, Emily P. Lane, Kelly Marnewick, Peter Caldwell, Glen Carlisle, Louw C. Hoffman

**Affiliations:** 1 Department of Ecosystem and Public Health, University of Calgary, Canada; 2 Department of Research and Scientific Services, National Zoological Gardens of South Africa, South Africa; 3 Carnivore Conservation Programme, Endangered Wildlife Trust, South Africa; 4 Old Chapel Veterinary Clinic, Pretoria, South Africa; 5 Oudtshoorn Veterinary Clinic, Oudtshoorn, South Africa; 6 Department of Animal Sciences, Stellenbosch University, South Africa

## Abstract

Whilst lead poisoning in raptors, scavenging birds and waterfowl is well studied and common knowledge, there is surprisingly little literature detailing the risk to mammalian scavengers and captive carnivores fed hunted meat. This case report describes the death of two captive cheetahs (*Acinonyx jubatus jubatus*) following acute onset of nervous symptoms. Clinical signs included hyper-excitability, seizures, arched back, tail held abnormally high and hyper-salivation. Necropsy findings included bullets or a bullet in their stomachs. Kidney and liver lead levels from one cheetah (15.6 ppm and 17 ppm respectively) were consistent with a diagnosis of lead poisoning; liver from the second cheetah was not available for testing. Both animals were routinely fed hunted antelope or game birds. This is the first report of oral lead poisoning in captive large carnivores, although these are unlikely to be the first cases. Without awareness of the risks of feeding hunted game, lead exposure will continue to be an underdiagnosed reality in the rehabilitation of endangered carnivores.

## Introduction

The southern African cheetah (*Acinonyx jubatus jubatus*) population declined significantly during the last century and the animal is listed as ‘vulnerable’ by the International Union for Conservation of Nature (Durant *et al.* 2008). Captive breeding and rehabilitation have been implemented to buffer declining wild population numbers. The intensive nature of these breeding projects has revealed numerous health problems, highlighting the importance of nutrition and stress management (Terio, Marker & Munson 2004).

Lead poisoning has long been recognised as a cause of illness and death (Chisolm 1971). Certain paints, lead storage battery plates, old pipes, industrial and mining waste, leaded petrol and lead shot are all typical sources of poisoning (Eisler 1988). Clinical cases of lead poisoning in livestock have occurred in Spain on farms on farmland around abandoned mines (Rodríguez-Estival, Pareja-Carrera & Mateo 2014). Waterfowl are at particular risk from the ingestion of shotgun pellets and fishing sinkers (Locke & Thomas 1996). Free-ranging predators and scavengers are exposed via poisoned waterfowl or gut piles left by hunters (Eisler 1988; Locke & Thomas 1996). Captive carnivores may ingest lead in animals shot for feeding purposes.

This case report describes two cases of lead toxicity in captive cheetahs due to ingestion of contaminated meat, and highlights the risks of feeding hunted meat to carnivores, even after removal of bullets.

## Case presentations

### Case 1

In November 2008 a two-year-old wild female cheetah from a temporary holding facility developed seizures, a raised tail and arched back. She died despite treatment and was submitted to the National Zoological Gardens of South Africa for necropsy examination.

The female cheetah was one of four young adults in a project tasked with re-establishing cheetah populations in protected, fenced reserves. To maximise survival orphaned young animals go through a re-wilding process to ensure they are fit and able to hunt effectively before being released. This is done by keeping the cheetah in a 1 ha enclosure initially. Antelope and birds are shot on the reserve as cheetah food. Once cheetahs are able to open and feed off a full carcass, they are released from the enclosure and monitored intensively. Management, handling and feeding schedules were consistent among the female and three males; the female was the only mortality.

Tissue preservation in the cheetah was poor due to delayed presentation. A representative sample of all tissues except eye and spinal cord were examined histologically. Macroscopic and histological features were non-specific and non-diagnostic. Four lead pellet fragments of less than one millimetre diameter were found in the stomach. Tissue lead concentrations of 15.6 ppm and 17 ppm (wet weight) for kidney and liver respectively were determined at the Onderstepoort Veterinary Institute Toxicology Department using atomic absorption spectrophotometry, and were highly suggestive of lead poisoning (Table 1). Organ sample and blanks were acid digested and compared with standard lead solutions.

**TABLE 1:** Published reference ranges for lead toxicity in selected species.

Species	Acute or chronic	ppm (wet weight)			ppm (dry weight)	
		Blood	Liver	Kidney	Bone	Hair
Domestic cats	Acute	0.30–2.90	10–73	-	-	-
Cattle	Acute	0.35–32.0	5.0–300	5.0–700	30–100	10–100
Dogs	Acute	0.60–8.60	50–200	10.0–50.0	-	> 88
Goats	Acute	0.90–1.00	> 10.0	> 10.0	-	-
Horses	Chronic	0.33–0.50	4.0–50	5.0–140	40–350	10–14
	Acute	0.60–2.50	10–500	20–200		5–10
Pigs	Acute	1.20–14.0	37–41	24–38	-	-
Chickens	Acute	4.00–12.0	18–90	20–150	> 400	-
Rabbits	Acute	2.80–4.00	> 10	> 10	-	-
Sheep	Acute	1.00–5.00	10–100	5.0–200	-	-
	Chronic	-	-	-	>70	> 25†
Geese	Acute	1.0–33.0	6–89	9–1600	3.3–33.0	10–100‡
Ducks / swans	Acute	3.3–33.0	8–137	8–992	-	-

*Source*: Adapted from Puls, R., 1994, *Lead. Mineral levels in animal health: Diagnostic data*, 2nd edn., Sherpa International, Clearbrook, BC

†, Wool; ‡, feathers (vane).

### Case 2

In April 2013 a seven-year-old male cheetah in a captive breeding centre became excited, started having seizures, became laterally recumbent and began to salivate excessively before dying under anaesthesia for examination. The cheetahs in this facility are kept for breeding purposes, housed in pairs in pens 0.4 ha – 1 ha in size, captive-born and hand-reared to facilitate management and reduce stress. Unlike most cases, this cheetah was wild-born and brought to the breeding centre as an adult 2 years prior to his death. He was housed alone due to his aggression to conspecifics. On the day of his death two males in an adjacent enclosure had been run along a passage between female enclosures to detect oestrus, eliciting excitement from all cheetahs in the vicinity.

The diet of cheetahs at this facility consists of meat from shot feral donkeys, horses, and occasionally rabbit, goat or chicken. This cheetah was fed large chunks of meat as his aggression precluded feeding minced meat in a dish. All cheetahs receive additional vitamin and mineral supplementation.

Necropsy revealed a lead bullet in the stomach. Tissues were submitted to the National Zoological Gardens of South Africa for histological examination, but not for lead determination. Histological findings were non-diagnostic and a presumptive diagnosis of lead poisoning was made.

## Discussion

In neither case could a definitive case of lead toxicity be established due to financial limitations, delay in submission of samples and submission of unsuitable samples for toxin testing. Timely submission of suitable samples for determination of lead and other toxins should be considered for any cases of neurological disease in captive cheetahs.

Added to this, lead tissue levels consistent with toxicity have not been published for cheetahs. There are surprisingly few reports of ingestion of lead in wild, non-avian carnivores: only three papers dealing with lead poisoning in large wild mammalian carnivores were found (Table 2). Tissue lead levels in the first case were of the same order of magnitude compared with those described for clinical lead toxicity in cattle (Ma 2011), horses (Dollahite *et al.* 1978) and mice (Clark 1979; Demayo *et al.* 1982), but higher than those described in domestic cats (Gilmartin *et al.* 1985). In all species lead toxicity depends on duration and dose of exposure (Table 1), making diagnosis of lead toxicity often presumptive rather than definitive (Eisler 1988). Risk of lead toxicity varies between species, and based on organic versus inorganic lead, maturity or life-stage, environmental temperatures, other dietary factors and level and duration of exposure to lead (Eisler 1988). Diagnosis is based on clinical and necropsy findings, microscopic organ changes and blood and tissue chemistry (Locke & Thomas 1996).

**TABLE 2:** Lead levels in liver and kidney of a captive cheetah with suspected lead toxicosis, with reported tissue levels for other species for reference.

Species	Captivity status	Exposure	Outcome	Stomach contents	Blood (µg/dL)	Liver (μg/g)†	Kidney (μg/g)†	Bone (μg/g)‡	Reference
Cheetah	Captive	Bullets / lead shot in game	Fatal	4 pellets < 1 mm	-	17	15.6	-	This study
Cougar	Wild	Bullets / lead shot in game or scavenged offal piles	Fatal	270 g of 2 mm – 3 mm lead shot, metal bullet jackets and glass shards	-	376	-	7.41	Burco *et al.* (2012)
Grizzly bear	Wild	Bullets / lead shot in game or scavenged offal piles	Subclinical monitoring	-	5.5 ± 4.0	-	-	-	Rogers *et al.* (2012)
Black bear	Wild			-	1.9 ± 1.2	-	-	-	
Iberian lynx	Wild	Mining waste and background soil concentrations	Subclinical monitoring	-	-	0.172 & 0.992	-	0.136 & 2.052	Millán *et al.* (2008)
Red fox	Wild			-	-	1.028	-	0.385	
Genet	Wild			-	-	0.184	-	0.112	
Egyptian mongoose	Wild			-	-	0.216	-	0.136	
Eurasian badger	Wild			-	-	1.84	-	0.735	
Horse	Domestic	-	Clinical toxicity	-	Depends on age and duration of exposure	18	16	-	Ma (2011)
Dog	Domestic	-	Clinical toxicity	-		23	32	735	
Cattle	Domestic	-	Clinical toxicity	-		26	50	-	
		-	Acute poisoning	-		16	> 32	-	
Cat	Domestic	Household objects, paint, etc.	Clinical toxicity	-	> 30–35	3.6–10	-	-	Knight and Kumar (2003)

Note: Please see the full reference list of the article, North, M.A., Lane, E.P., Marnewick, K., Caldwell, P., Carlisle, G. & Hoffman, L.C., 2015, ‘Suspected lead poisoning in two captive cheetahs (*Acinonyx jubatus jubatus*) in South Africa, in 2008 and 2013’, *Journal of the South African Veterinary Association* 86(1), Art. #1286, 5 pages. http://dx.doi.org/10.4102/jsava.v86i1.1286, for more information.

†, Liver and kidney lead concentrations are on a wet weight (ww) basis; conversion factors of 6.5 for kidney and 4.0 for liver (Ma 2011) were used to calculate ww from dry weight (dw); ‡, bone lead concentration is on a dw basis.

In these cheetahs typical pathological signs of lead toxicity, such as cerebral oedema and necrosis, peripheral nerve Wallerian degeneration, renal intranuclear inclusions and anaemia (Haschek, Rousseaux & Wallig 2010) were either absent or obscured by autolysis. However, since lead is neither essential nor beneficial to living organisms and all existing data indicate that its metabolic effects are adverse (Eisler 1988), the absence of indications of degenerative, infectious, traumatic or parasitic disease supported a diagnosis of lead toxicity.

No histological lesions consistent with viral encephalitis were observed in either case. Inadvertent poisoning with other toxicants (pesticides, etc.) could not be definitively ruled out in either cheetah. However, since both cheetahs were housed in captive environments where access to food and water was controlled and the enclosures were clean and well-maintained, exposure to other toxicants was considered unlikely. Environmental exposure to toxicants or infectious disease would be expected to affect multiple animals, particularly those housed with or adjacent to the affected cheetahs, or fed the same food as the affected cheetahs. The accidental ingestion of a single lead bullet or shot is more likely to affect individual animals, and was therefore top of the list of possible differential diagnoses.

Whilst the presence of a lead bullet or shot in an animal's stomach may not provide proof of lead poisoning, it does prove that the animal has been exposed to lead, and that this may not have been the first time. The gastro-intestinal stasis commonly associated with lead intoxication may increase the retention time (Locke & Thomas 1996), whilst highly acidic stomach content promotes lead dissolution and absorption of lead salts (Pain 1996; Pain *et al.* 2007). Pattee *et al.* (1981) demonstrated experimentally that acute exposure of bald eagles to lead shot can result in death within as little as 10 days, although with wide variation between individual birds.

Lead is a cumulative, multisystemic metabolic toxin (Eisler 1988). The earliest indicator of exposure is the blood lead concentration, which has a half-life of approximately 1 month after acute exposure. Soft tissues such as liver and kidney may maintain lead levels for approximately 2 months after cessation of exposure (Rabinowitz, Wetherill & Kopple 1976). Lead half-life varies, depending on exposure route and whether lead has reached steady-state level in the animal (as occurs with chronic exposure) (Ma 2011). A significant body of literature is available on the effects of lead intoxication on waterfowl, scavenging and predatory birds (Blus *et al.* 1995; Gangoso *et al.* 2009; Lambertucci *et al.* 2011; Lewis *et al.* 2001; Nadjafzadeh, Hofer & Krone 2013; Pain *et al.* 2007). Similarly, human exposure (Castiglia 1995; Waldron 1975; Waldron, Mackie & Townshend 1976) and exposure of domestic livestock to lead (Roegner *et al.* 2013; Sharma *et al.* 1982) are well documented.

Extensive disintegration and dissemination of microscopic lead particles along the wound tract provide a source of exposure for scavenging birds and mammals feeding on offal piles (Hunt *et al.* 2006). Pauli and Buskirk (2007), using a combination of gross fragment extraction and flame atomic absorption spectrometry, found a mean whole-body lead content of 228.4 mg in 10 black-tailed prairie dog (*Cynomys ludovicianus*) carcasses that had been shot using expanding lead bullets. This is concerning, as a single carcass shot recreationally could potentially acutely poison scavengers.

Importantly, death may not be the only outcome of toxicity. Rogers *et al.* (2012) demonstrated that specific free-ranging wild species in the Yellowstone area currently carry lead burdens that may be sufficiently high to affect their survival and reproduction. Worst affected are scavenging species that regularly consume hunters’ leavings: wounded game or offal piles. Unfortunately not enough is known about the sensitivity of these species to predict the consequences of their current lead burden. These two cases serve as a reminder that subclinical lead toxicity could affect survival and reproduction in captive cheetahs.

In South Africa carnivores are managed in a network of small fenced reserves (e.g. lions [Miller *et al.* 2013], African wild dogs [Davies-Mostert, Mills & MacDonald 2009] and cheetahs [Marnewick *et al.* 2009]). Careful management is therefore necessary to prevent inbreeding and overpopulation in these reserves, frequently requiring animals to be kept in small enclosures whilst being moved between reserves or when artificially forming social groups. In temporary holding these carnivores are fed on game meat that is generally sourced from the reserve and shot with a rifle.

## Conclusion

Lead poisoning is an understudied and potentially underdiagnosed condition of captive and wild carnivores. In this context it represents a risk to the health and welfare of any captive carnivores, including avian, being fed hunted game. With so many rare or endangered species being maintained in captive facilities, the risk of reducing their viability by exposing them to lead is unnecessary and preventable.

It is suggested that careful bullet removal and extensive wound tract excision, or ideally the use of non-lead ammunition for shooting food animals, become key husbandry recommendations for captive carnivores in temporary holding. Additionally, whilst lead poisoning should always be high on the list of differential diagnoses for captive carnivores with neurological symptoms or sudden death, it is recommended that good-quality fresh tissue samples be submitted to a pathologist as soon as possible to rule out viral encephalitis and exposure to other toxins.
